# Humate-assisted Synthesis of MoS_2_/C Nanocomposites via Co-Precipitation/Calcination Route for High Performance Lithium Ion Batteries

**DOI:** 10.1186/s11671-018-2537-y

**Published:** 2018-04-27

**Authors:** Qin Geng, Xin Tong, Gideon Evans Wenya, Chao Yang, Jide Wang, A. S. Maloletnev, Zhiming M. Wang, Xintai Su

**Affiliations:** 1 Institute of Fundamental and Frontier Science, University of Electronic Science and Technology of China, Chengdu, 610054 People’s Republic of China; 2 Ministry Key Laboratory of Oil and Gas Fine Chemicals, College of Chemistry and Chemical Engineering, Xinjiang University, Urumqi, 830046 People’s Republic of China; 3 Moscow State Mining University, Moscow, 109807 Russia

**Keywords:** MoS_2_/C nanocomposites, Humate, Co-precipitation/calcination route, Anode, Lithium-ion batteries

## Abstract

A facile, cost-effective, non-toxic, and surfactant-free route has been developed to synthesize MoS_2_/carbon (MoS_2_/C) nanocomposites. Potassium humate consists of a wide variety of oxygen-containing functional groups, which is considered as promising candidates for functionalization of graphene. Using potassium humate as carbon source, two-dimensional MoS_2_/C nanosheets with irregular shape were synthesized via a stabilized co-precipitation/calcination process. Electrochemical performance of the samples as an anode of lithium ion battery was measured, demonstrating that the MoS_2_/C nanocomposite calcinated at 700 °C (MoS_2_/C-700) electrode showed outstanding performance with a high discharge capacity of 554.9 mAh g^− 1^ at a current density of 100 mA g^− 1^ and the Coulomb efficiency of the sample maintained a high level of approximately 100% after the first 3 cycles. Simultaneously, the MoS_2_/C-700 electrode exhibited good cycling stability and rate performance. The success in synthesizing MoS_2_/C nanocomposites via co-precipitation/calcination route may pave a new way to realize promising anode materials for high-performance lithium ion batteries.

**Electronic supplementary material:**

The online version of this article (10.1186/s11671-018-2537-y) contains supplementary material, which is available to authorized users.

## Background

Due to their high energy density, long cycle life, and environmental friendliness, lithium ion batteries (LIBs) are widely utilized in portable electronic devices [1] (e.g., mobile phones and watches), electric vehicles [2, 3], and renewable energy storage [4–8]. Graphite is the most widely used anode materials in commercial LIBs, benefiting from its low working voltage, good conductivity, and low cost [9–11]. However, the characteristic structure of graphite leads to feasible generation of LiC_6_, allowing only one lithium ion intercalation in every six carbon atoms which results in a low theoretical specific capacity of 372 mAh g^− 1^, which is far away from the current business requirements [12].

Currently, it is preferred to obtain appropriate electrode materials in LIBs for higher battery capacity, longer cycle life, and better rate capability. Consequently, Li-alloy-based anode materials [13], transition metal oxides [14], oxysalts, and transition metal sulfides [15] are often served as the anode materials in LIBs, since these materials display all the necessary properties for appropriate electrode materials. Among these materials, transition metal sulfides (e.g., CuS_2_ [16], WS_2_ [17], and MoS_2_ [18–20] have been an exciting topic in research as they are earth-abundant and show high specific capacity when used as anode materials in LIBs [21]. As a typical representative, MoS_2_ has gained a lot of attention due to its particular S-Mo-S layered structure [22], high theoretical specific capacity compared to traditional graphite anode, and there is a transfer reaction of four electrons when used as anode materials in LIBs [23, 24]. In addition, the van der Waals forces between the MoS_2_ layers are very weak, allowing lithium-ion diffusion without causing significant volume change [25, 26]. However, MoS_2_ is still an unsatisfactory anode material due to its low electrical conductivity, leading to the poor cycling and rate performance [27]. To solve this problem, a number of strategies have been developed to improve its electrical conductivity such as the incorporation of MoS_2_ with carbon materials [28–30].

To date, a variety of MoS_2_/carbon composites have been synthesized as anode materials in LIBs, namely, layered MoS_2_/graphene composites [31], MoS_2_/C multilayer nanospheres [32], MoS_2_-CNT composite [33], multilayered graphene/MoS_2_ heterostructures [34], or petal-like MoS_2_ nanosheets space-confined in hollow mesoporous carbon spheres [35]. Despite gratifying progress in electrical conductivity, cycling, and rate performance of the electrode, some other conflicts in the synthesis method have persisted. At present, the most commonly used synthetic method is hydrothermal approach followed by an annealing process, which can introduce carbon matrix with some surfactants such as sodium oleate or oleyamine and sulfur element with some L-cysteine in the first procedure. Moreover, expensive and toxic organic reagents were always indispensable and unavoidable during the synthesis process when compared with co-precipitation method. Currently, co-precipitation method is just beginning to gain popularity in the synthesis of inorganic nanostructured materials due to its cost-effective, non-toxic, trustworthy, and stable [36, 37]. To the best of our knowledge, there has been little report on the synthesis of MoS_2_/C nanocomposite by co-precipitation/calcination process, especially with potassium humate.

Potassium humate, a sort of aromatic hydroxy carboxylate, which consisted of a wide variety of oxygen-containing functional groups, can be considered as functionalized graphene candidate [38]. In general, a great deal of researches have been made to use potassium humate as carbon source to synthesize carbon materials under extremely harsh conditions [38, 39]. Huang [38] reported that potassium humate can be straightforward carbonization to prepare reduced graphite oxide materials. In this paper, MoS_2_/C nanocomposites were synthesized via a co-precipitation/calcination route, by employing an organic matter (potassium humate) and an inorganic substance ((NH_4_)_6_Mo_7_O_24_) as reagents. The electrochemical performance of the samples as a LIBs anode was measured, and the results showed that the sample calcinated at 700 °C (MoS_2_/C-700) exhibited better cycling ability and rate behavior. The discharge capacity of the sample remained at 554.9 mAh g^− 1^ after 50 cycles at the current density of 100 mA g^− 1^, which is much better than the other two samples calcinated at 600 °C and 800 °C, respectively. Meanwhile, the as-prepared MoS_2_/C-700 displays a comparable electrochemical performance [40, 24].

## Methods/Experimental

Potassium humate was obtained from Double Dragons Humic Acid Co., Ltd. Xinjiang (China), and the composition analysis of potassium humate was shown in Additional file 1: Table S1. All of the chemical reagents (except potassium humate) were of pure analytical grade and used without further purification.

### Synthesis of MoS_2_/C

The precursor was prepared by co-precipitation from (NH_4_)_6_Mo_7_O_24_ and potassium humate in the presence of HNO_3_ followed by a freeze-dried process for 2 days. In a typical procedure, 4 g of potassium humate were dissolved in 40 mL of 0.25 M (NH_4_)_6_Mo_7_O_24_ solution. Subsequently, the above-mentioned solution was added dropwise to 100 mL of 0.5 M HNO_3_ solution with vigorous magnetic stirring. The duration of the magnetic stirring was for several hours. The lower precipitation was then separated from the mixture solution, freeze-dried, and labeled as Mo-HA precursor. The precursor was mixed with anhydrous Na_2_SO_4_ (with a proportion of 1:10) and ground in a mortar to form a homogeneous mixture. The mixture was then calcinated at 700 °C for 3 h (with a heating rate of 10 °C min^− 1^) and then naturally cooled down to room temperature. Finally, the products were washed with deionized water and ethanol three times followed by a freeze-dried procedure to obtain the MoS_2_/C powder. In parallel, the samples calcinated at 600 and 800 °C were synthesized as well.

### Characterization

The surface organic functional groups of potassium humate were measured by Fourier transform spectrophotometer (FT-IR, VERTEX 70, Bruker) with KBr as the reference sample. The structure and morphology of different samples were characterized by X-ray diffraction (XRD, BRUKER D8 Advance) with Cu Kα radiation (λ = 1.54178 Å), transmission electron microscopy (TEM, Hitachi H-600), high-resolution transmission electron microscopy (HRTEM, JEM-2100F), LEO 1450VP scanning electron microscope (SEM), energy-dispersive X-ray spectrometer (EDX), and X-ray photoelectron spectroscopy (XPS, ESCALAB 250Xi spectrometer). Thermogravimetric analyses (TGA) were conducted on a thermogravimetric analyzer (Netzsch TGA 409). Raman spectrum was carried out on Bruker Senterra with 532 nm wavelength.

### Electrochemical Measurements

Electrochemical measurements were performed on coin cells. The working electrodes were fabricated by mixing 80 wt.% of the as-prepared MoS_2_/C active materials, 10 wt.% of acetylene black, and 10 wt.% of polyvinylidene fluoride (PVDF) in N-methyl-2-pyrrolidinone (NMP) solvent to form a homogeneous slurry. The slurry was coated on the copper foil and dried in a vacuum at 110 °C for 12 h. The coin cells were assembled in an argon-filled glovebox. In the measurement, lithium foil was used as the counter electrode and reference electrode, and a polypropylene film (Celgard-2400) was used as a separator. The electrolyte solution was 1 mol L^− 1^ LiPF_6_ in ethylene carbonate (EC), dimethyl carbonate (DMC), and diethyl carbonate (DEC) (EC/DMC/DEC, 1:1:1, volume ratio). The galvanostatic charge-discharge measurements were performed in a potential range of 0.01–3.0 V by using a LAND CT2001A battery testing instrument (Wuhan) at room temperature. Cyclic voltammetry (CV) measurements were performed on an electrochemical work-station (CHI 660D) at a scanning rate of 0.1 mV s^− 1^ between 0.01 and 3.0 V.

## Results and Discussion

**Fig. 1:**
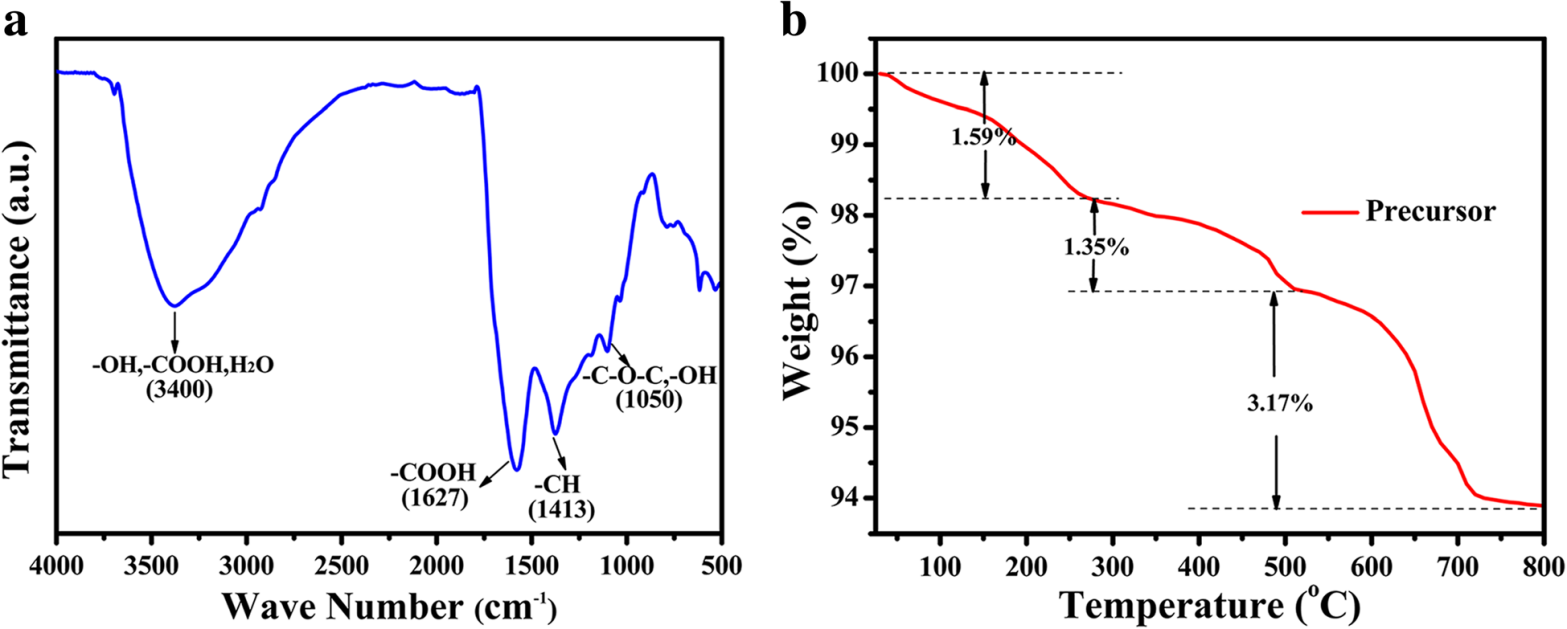
**a** FT-IR spectra of pure potassium humate. **b** TGA curve of the homogeneous mixture of Mo-HA precursor and anhydrous Na_2_SO_4_ (with a proportion of 1:10)

The surface chemistry of potassium humate was studied using FTIR spectrum. In Fig. 1a, the broad peaks centered at 3400 cm^− 1^ were ascribed to the stretching vibrations of −OH, −COOH, and H_2_O bonds, The peaks at 1627, 1413, and 1050 cm^− 1^ were attributed to the stretching vibrations of the −COO groups and −CH, −OH and so on [41], respectively, indicating the rich oxygen-containing functional groups on the surface of pure potassium humate, which is beneficial to complexation reaction or adsorption. TGA curve of the homogeneous mixture of Mo-HA precursor and anhydrous Na_2_SO_4_ (with a proportion of 1:10) in an argon atmosphere with a heating rate of 10 °C min^− 1^ is shown in Fig. 1b. It can be seen that there are three steps of weight loss in the TGA curve. The first weight loss is 1.59% from room temperature to 250 °C, which may be due to decomposition of the water in the surface of the Mo-HA precursors. There are another two consecutive steps of weight loss, with a weight loss of 1.35% from 250 to 500 °C, and a weight loss of 3.17% from 500 to 800 °C, and then the mass remains constant, indicating that the precursor has been decomposed completely at 800 °C. For such a system, we choose those three temperatures for calcination as 600, 700, and 800 °C, denoted as MoS_2_/C-600, MoS_2_/C-700, and MoS_2_/C-800, respectively.

**Scheme 1:**
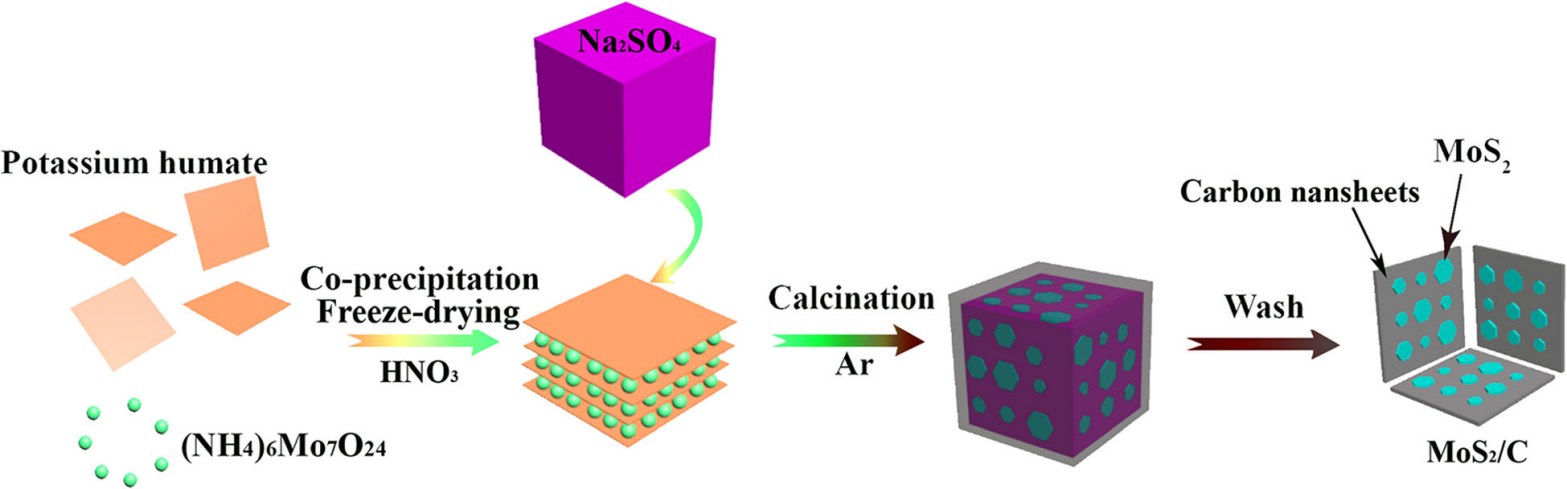
Schematics depicting the fabrication procedure of MoS_2_/C nanocomposite

According to the literature [34], a possible mechanism of the reaction process has been proposed and schematically depicted in Scheme 1. Moreover, the corresponding formulas are listed in Additional file 1: Equations 1–5. In these equations, potassium humate was abbreviated as K-HA. There might be a complexation when potassium humate was dissolved in (NH_4_)_6_Mo_7_O_24_ solution, with the participation of HNO_3_ solution, which leads to the generation of Mo-HA. After heating the mixture of the Mo-HA precursor and anhydrous Na_2_SO_4_ in an argon atmosphere at a relatively high temperature, the Mo-HA precursor would be carbonized to form the intermediate of amorphous carbon, and then the intermediate would reduce anhydrous Na_2_SO_4_ to generate Na_2_S, further hydrolyzed to hydrogen sulfur. Finally, hydrogen sulfur may react with MoO_x_, leading to the formation of MoS_2_/C nanocomposites.

**Fig. 2:**
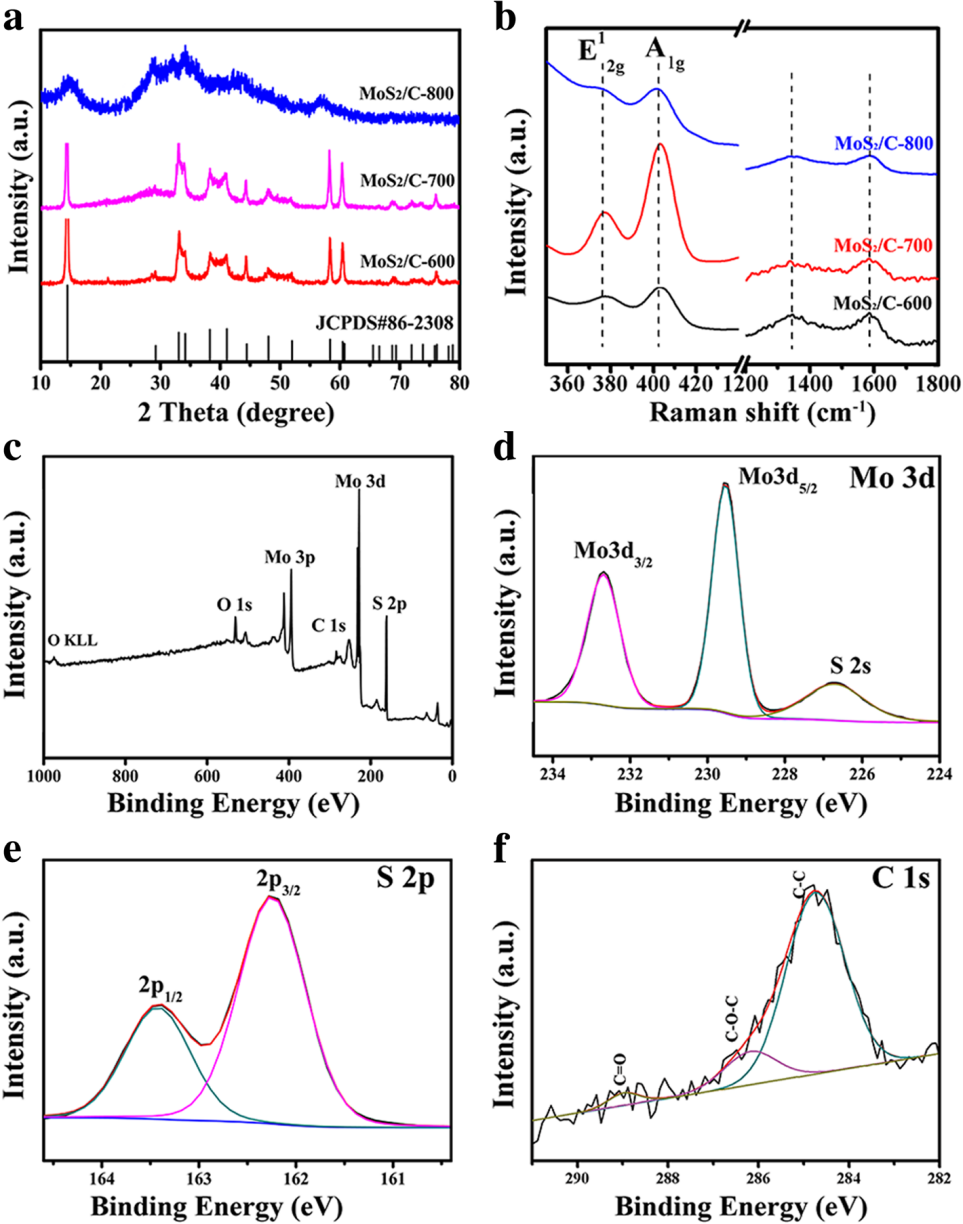
**a** XRD patterns. **b** Raman spectra of MoS_2_/C nanocomposites calcinated at different temperatures. **c** Survey XPS spectra of MoS_2_/C-700. **d** High-resolution XPS spectra of Mo 3d. **e** S 2p. **f** C 1 s

Figure 2a–b show the XRD patterns and Raman spectra of the MoS_2_/C nanocomposites calcinated at different temperatures. Figure 2a shows that almost all the diffraction peaks of MoS_2_/C-600 and MoS_2_/C-700 can be well indexed to the hexagonal MoS_2_ phase (JCPDS card no. 86-2308), which is consistent with those of previous report [42]. There are still some other peaks mismatching the standard card in the MoS_2_/C-800 sample. We speculate that the crystalline of MoS_2_/C has been destroyed at high temperature. From the Raman spectra (Fig. 2b), it can be seen that the peaks located in between 379 and 400 cm^− 1^ belonged to E^1^_2g_ (the in-plane displacement of Mo and S atoms) and A _1g_ (out-of-plane symmetric displacement of Mo and S atoms) Raman modes, respectively [24, 43]. The bands appeared at 1347 and 1589 cm^− 1^ were the characteristic D- and G-band, and the value of *I*_*D*_/*I*_*G*_ were 0.96, 0.91, and 0.94 as the temperature goes from 600 to 800 °C. The former corresponds to the amorphous carbon or sp^3^-hybridized carbon (D-band), and the latter assigned to the sp^2^-hybridized carbon (G-band) [44]. Although there is no great distinction between the degree of graphitization, the MoS_2_/C-700 sample is still a little higher than the other two samples to a certain extent, indicating that the carbon in this sample is not only in the form of amorphous carbon, but also some graphitic carbon. Therefore, we focused on the MoS_2_/C-700 sample in the following investigations.

To further study the chemical composition and chemical bonds of MoS_2_/C-700, X-ray photoelectron spectroscopy (XPS) analysis was carried out. The survey XPS spectrum (Fig. 2c–f) reveals the presence of Mo, S, C, and O elements in the MoS_2_/C-700 nanocomposite. The high-resolution XPS spectra of Mo 3d and S 2p are shown in Fig. 2d, e, respectively. The peaks at 229.4 and 232.6 eV are assigned to the Mo 3d_5/2_ and Mo 3d_3/2_, confirming the existence of Mo in MoS_2_/C-700 [45, 46]. The presence of another XPS peak at 226.5 eV is indexed to S 2 s, which is resulted from the surface of the MoS_2_/C-700 [47]. Moreover, the XPS peaks at 162.3 and 163.4 eV in S 2p spectra are characteristic peaks of the S 2p_3/2_ and S 2p_1/2_ of MoS_2_, respectively. Figure 2f shows that the C1 s spectrum can be divided into three peaks, denoted as C–C, C–O, and C=O groups, respectively.

**Fig. 3:**
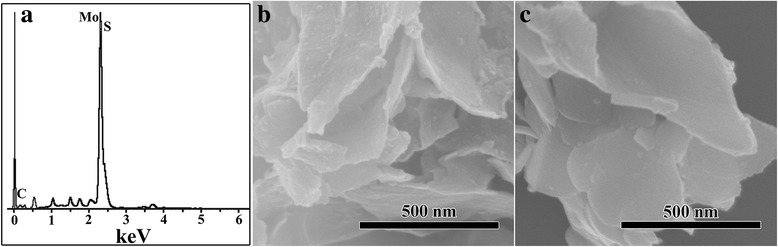
**a** EDX spectrum of MoS_2_/C-700. **b**, **c** SEM images of MoS_2_/C-700 nanocomposite

**Fig. 4:**
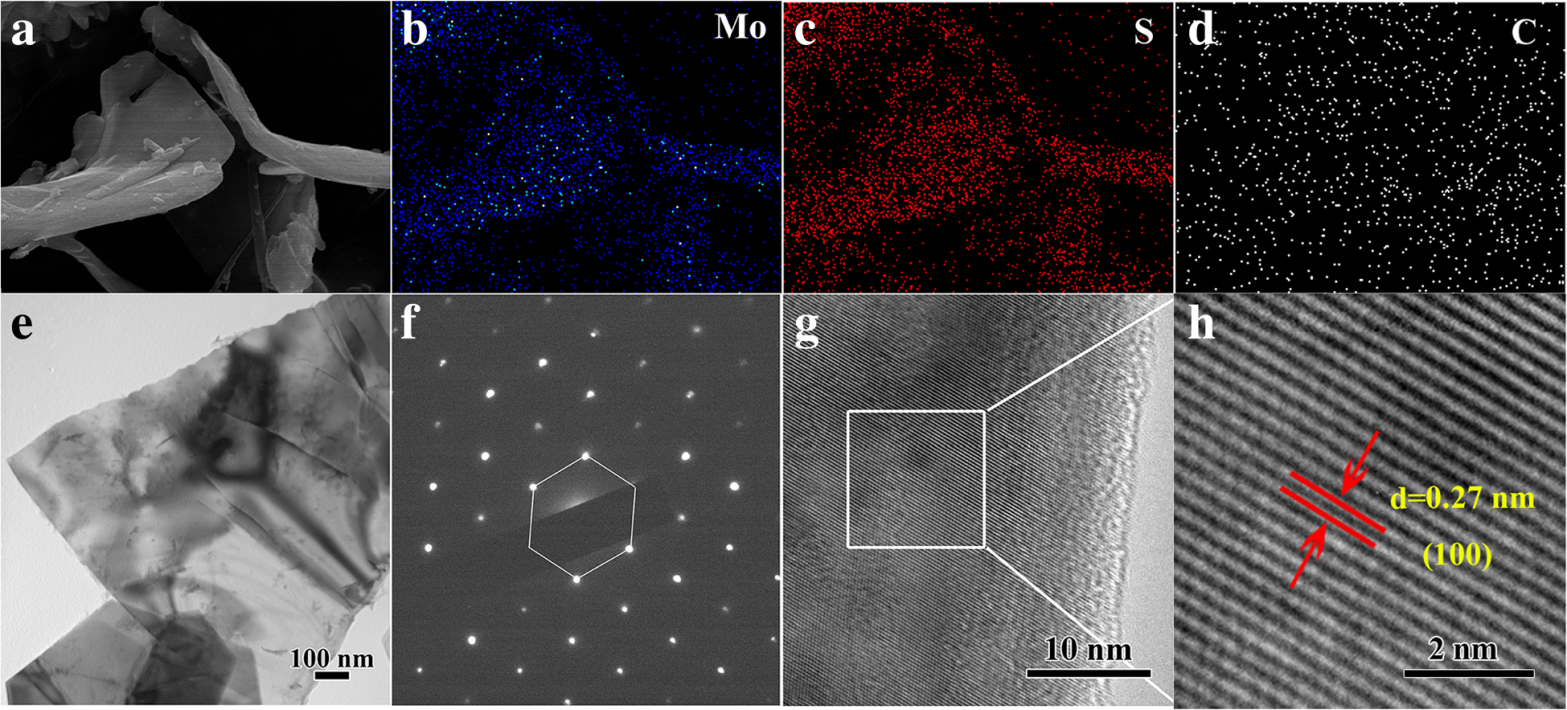
**a**-**d** Elemental mapping images of MoS_2_/C-700; (**e**) TEM image, (**f**) the SAED and (**g**) High resolution TEM image of MoS_2_/C-700 nanocomposite, (**h**) Enlarged HR-TEM image of the marked area in figure (**g**)

The EDX spectrum indicates that the sample calcinated at 700 °C contains Mo, S, and C elements, as shown in Fig. 3a. Figure 3b, c show the SEM images of the sample of MoS_2_/C-700. For comparison, the SEM images of MoS_2_/C-600 nanocomposite and MoS_2_/C-800 nanocomposite were also shown in Additional file 1: Figure S1. In order to explore the corresponding element distribution in the sample of MoS_2_/C-700, the corresponding elemental mapping analysis were carried out. As shown in Fig. 4a–d, the elemental mapping images of MoS_2_/C-700 demonstrated the uniform distribution of Mo, S, and C all over the MoS_2_/C-700 nanocomposite, which is consistent with the EDX and XPS results.

As displayed in Fig. 4e–h, the morphology and structure of the as-synthesized MoS_2_/C nanocomposites were investigated by transmission electron microscopy (TEM), selected area electron diffraction (SAED), and high-resolution transmission electron microscopy (HRTEM). The TEM image (Fig. 4e) and the SEM images (Fig. 3b, c) clearly show that the structure of MoS_2_/C-700 nanocomposite is wrinkled two-dimensional nanosheets with the width of ~ 800 nm and the thickness of ~ 20 nm. SAED pattern in Fig. 4f shows that the hexagonal lattice structure of MoS_2_ is well crystallized. Furthermore, the crystal lattices of the sample were shown at HRTEM profiles ((Fig. 4g, h) and Additional file 1: Figure S2). The profiles showed highly crystalline MoS_2_ nanosheets with an interlayer distance of 0.27 nm corresponding to (100) plane of hexagonal MoS_2_ [24, 34]. In addition, Additional file 1: Figure S2 clearly reveals that the carbon nanosheets were decorated with MoS_2_ nanosheets.

**Fig. 5:**
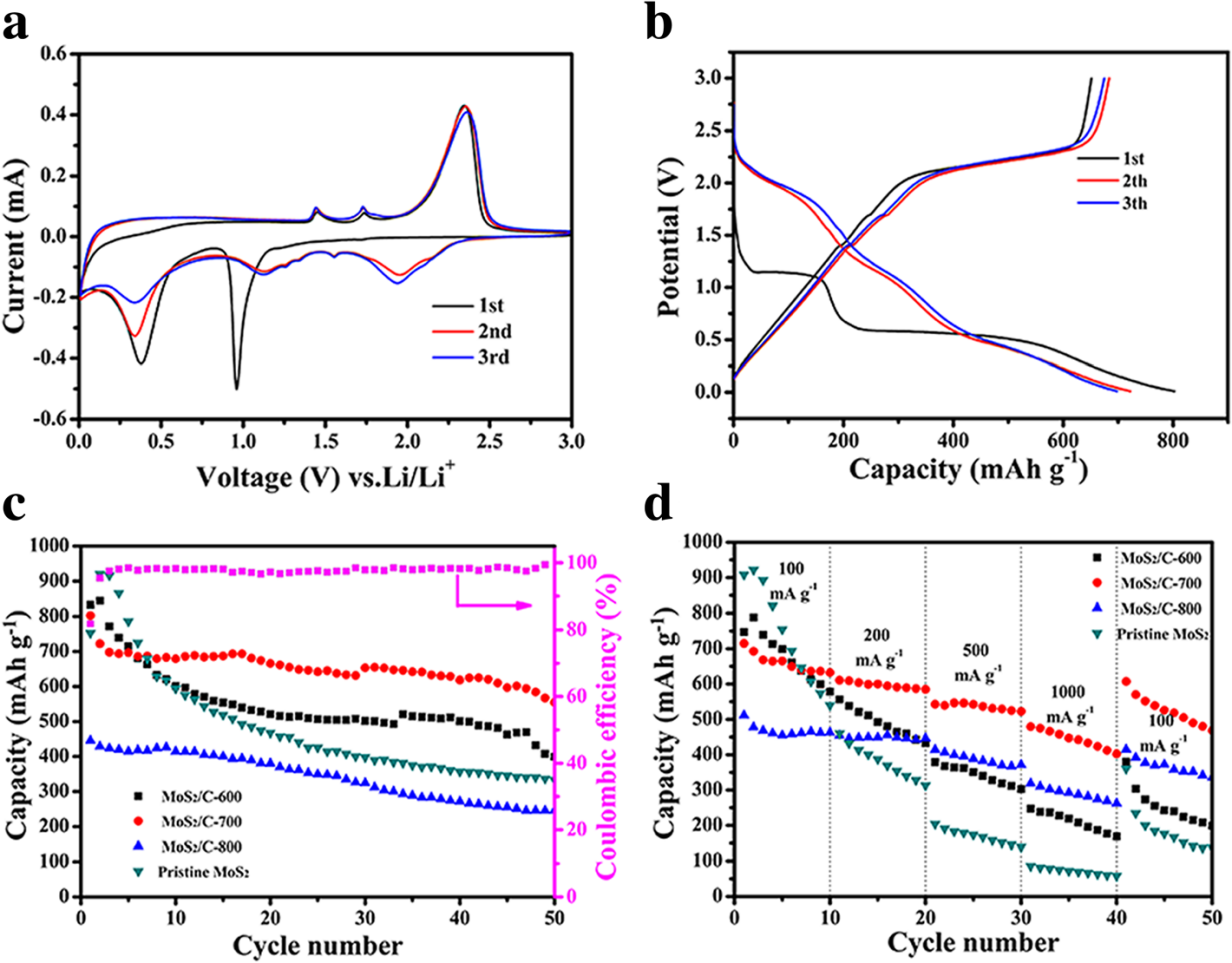
**a** CV curves of the first three cycles of MoS_2_/C-700 electrode at a scan rate of 0.1 mV s^− 1^. **b** Discharge and charge curves of the first 3 cycles of MoS_2_/C-700 electrode at a current density of 100 mA g^− 1^. **c** Cycling performance MoS_2_/C electrode and the pristine MoS_2_ electrode at a current density of 100 mA g^− 1^, and Coulombic efficiency of MoS_2_/C-700 electrode. **d** Rate performance of MoS_2_/C and the pristine MoS_2_ electrode at the current densities ranging from 100 to 1000 mA g^− 1^

Figure 5a shows the CV curves of the first 3 cycles of MoS_2_/C-700 electrode at a scan rate of 0.1 mV s^− 1^ in the potential window of 0.01–3.00 V vs. Li^+^/Li. During the first cycle, the reduction peak at 1.0 V indicates the lithium insertion mechanism, which is due to the insertion of lithium ions into the MoS_2_ layers to form Li_x_MoS_2_. At the same time, there has been a phase transition from 2H (trigonal prismatic) to 1T (octahedral) [48]. Another reduction peak at 0.4 V is attributed to the conversion of Li_x_MoS_2_ into metallic Mo and Li_2_S. The broad oxidation peak located at 2.35 V represents the deintercalation of Li_2_S to S. During the subsequent cycles, the two cathodic peaks at 1.0 and 0.4 V disappear with appearance of three new peaks at 2.0, 1.2, and 0.3 V, indicating the reduction of MoS_2_ and the conversion from S_8_ to polysulfides and then to Li_2_S [24].

The discharge and charge curves of the first 3 cycles of MoS_2_/C-700 electrode were recorded, and the corresponding results were shown in Fig. 5b. In the first cycle, the discharge and charge capacities of MoS_2_/C-700 electrode are 802.8 and 651.4 mAh g^− 1^, respectively, with a Coulomb efficiency of 81.14%. The irreversible capacity loss may be due to some irreversible reaction such as the decomposition of electrolyte and the formation of solid electrolyte interface (SEI) film [49, 50].

The cycle stability of whole MoS_2_/C electrode and the pristine MoS_2_ electrode at a current density of 100 mA g^− 1^ are presented in Fig. 5c. At the same time, the Coulomb efficiency of MoS_2_/C-700 is also recorded. After 50 cycles, the discharge capacities of MoS_2_/C-600, MoS_2_/C-700, MoS_2_/C-800, and pristine MoS_2_ electrode at a current density of 100 mA g^− 1^ remain at 399.7, 554.9, 245.7, and 332.9 mAh g^− 1^, respectively. As shown in Additional file 1: Table S1, it has summarized the discharge capacities after 50 cycles of MoS_2_-based electrode presented in other literature, the as-prepared MoS_2_/C-700 display a comparable electrochemical performances compared to the previous work. It is concluded that the MoS_2_/C-700 electrode shows the most outstanding cycle performance and the Coulomb efficiency of the sample maintained a high level at about 100% after the first 3 cycles. It may benefit from the small amount of graphitic carbon in this sample, leading to enhanced electrical conductivity of the nanocomposite.

In addition to the cycling stability, the high-rate performance is also an important factor for high-power applications. Figure 5d shows the rate performance of MoS_2_/C and the pristine MoS_2_ electrode at the current densities ranging from 100 to 1000 mA g^− 1^. At 1000 mA g^− 1^, the discharge capacity of MoS_2_/C-700 can still maintain at a relatively high value of ~ 450 mAh g^− 1^, which is higher than the other MoS_2_/C electrodes and pristine MoS_2_ electrode we have prepared at the same current density. When the current density is changed back to 100 mA g^− 1^, the capacity of MoS_2_/C-700 sample can recover up to ~ 500 mAh g^− 1^ after 50 cycles at different current densities, revealing the good rate capability of the sample.

**Fig. 6:**
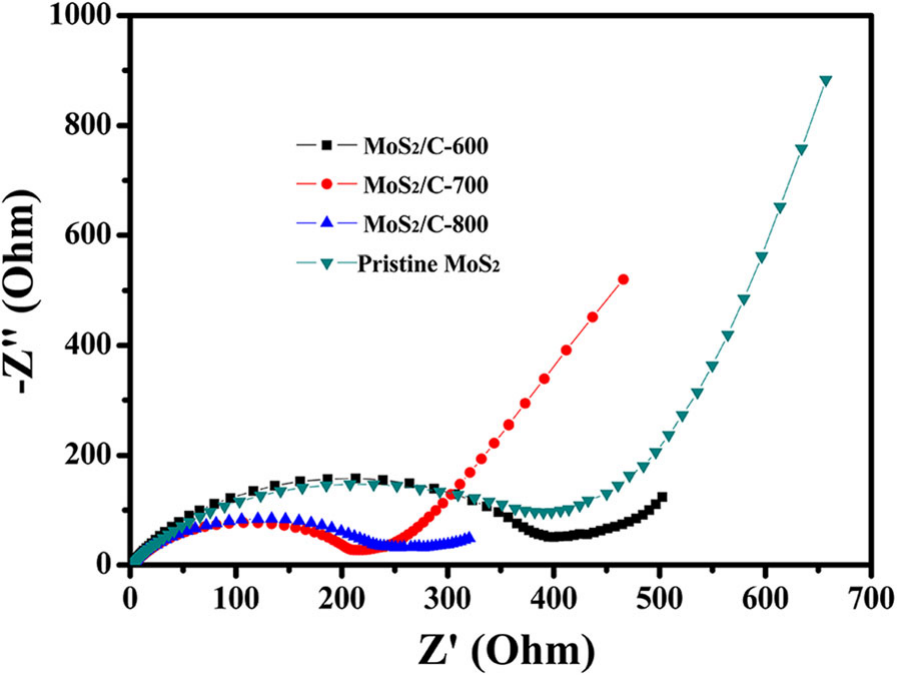
Nyquist plots of the MoS_2_/C electrode and the pristine MoS_2_ electrode tested in a frequency range of 0.01 Hz to 100 kHz

The electrochemical impedance spectra (EIS) measurements on the MoS_2_/C and the pristine MoS_2_ electrode were conducted in order to gain a further understanding about the excellent electrochemical performance of the MoS_2_/C-700 sample (Fig. 6). There is a semicircle at the high frequency region followed by a slope line at the low frequency region on these Nyquist plots. It can be seen that the semicircle at the high frequency region of the MoS_2_/C-700 sample is evidently smaller than that of the other three samples, which is related with the charge transfer resistance (*R*_ct_) occurred at the electrolyte and electrodes interfaces. Therefore, this result further implies that the incorporation of potassium humate markedly improve the conductivity of MoS_2_, leading to further improvement in the electrochemical performances.

## Conclusions

In this work, two-dimensional MoS_2_/C nanosheets were successfully synthesized via a co-precipitation/calcination route by employing an organic matter (potassium humate) and an inorganic substance ((NH_4_)_6_Mo_7_O_24_) as reagents. Structural characterizations show that as-prepared MoS_2_/C-700 nanocomposite is two-dimensional (2D) MoS_2_/C nanosheets with irregular shape. The 2D MoS_2_/C nanosheets exhibited improved electrochemical performance when fabricated as anode material for LIBs. Furthermore, a possible reaction process was proposed. The current synthesis strategy may be expanded into the synthesis of other nanocomposite that can be served as anode materials for high-performance lithium-ion batteries.

## Additional file

Additional file 1: **Equations 1–5.** The proposed reactions for the synthesis of MoS_2_. **Table S1.** The composition analysis of potassium humate. **Figure S1.** SEM images of (a) MoS_2_/C-600 and (b) MoS_2_/C-800 nanocomposite. **Figure S2.** High-resolution TEM image of MoS_2_/C-700 nanocomposite. **Table S2.** Comparison of electrochemical performance of MoS_2_-based electrodes. (DOC 2203 kb)

## Abbreviations

2D: Two-dimensional
CV: Cyclic voltammetry
DEC: Diethyl carbonate (DEC)
DMC: Dimethyl carbonate
EC: Ethylene carbonate
EDX: Energy-dispersive X-ray spectrometer
EIS: Electrochemical impendence spectroscopy
FT-IR: Fourier transform spectrophotometer
HRTEM: High-resolution transmission electron microscopy
*I*_*D*_: The intensity of D-band
*I*_*G*_: The intensity of G-band
LIBs: Lithium ion batteries
Mo-HA: The precursors
MoS_2_/C: MoS_2_/carbon
MoS_2_/C-600: MoS_2_/C nanocomposite calcinated at 600 °C
MoS_2_/C-700: MoS_2_/C nanocomposite calcinated at 700 °C
MoS_2_/C-800: MoS_2_/C nanocomposite calcinated at 800 °C
NMP: N-methyl-2-pyrrolidinone
PVDF: Polyvinylidene fluoride
*R*_ct_: Charge transfer resistance
SEM: Scanning electron microscope
TEM: Transmission electron microscopy
TGA: Thermogravimetric analyses
XRD: X-ray diffraction
