# Supplementary material for: Humate-assisted Synthesis of MoS2/C Nanocomposites via Co-Precipitation/Calcination Route for High Performance Lithium Ion Batteries
Source: Nanoscale Res Lett. 2018 Apr 27;13:129. doi: 10.1186/s11671-018-2537-y (PMC5924512; doi:10.1186/s11671-018-2537-y)
Supplement: Supplementary file 1 — Equations 1–5. The proposed reactions for the synthesis of MoS2. Table S1. The composition analysis of potassium humate. Figure S1. SEM images of (a) MoS2/C-600 and (b) MoS2/C-800 nanocomposite. Figure S2. High-resolution TEM image of MoS2/C-700 nanocomposite. Table S2. Comparison of electrochemical performance of MoS2-based electrodes. (DOC 2203 kb) [file 11671_2018_2537_MOESM1_ESM.doc]

**Additional file 1**

**Humate-assisted Synthesis of MoS_2_/C Nanocomposites via** **Co-precipitation/calcination Route for High Performance Lithium Ion Batteries**

Qin Geng ^[a,b]^, Xin Tong ^[a]^, Gideon Evans Wenya ^[a]^, Chao Yang ^[b]^, Jide Wang ^[b]^, A. S. Maloletnev ^[c]^, Zhiming M. Wang *^[a]^, Xintai Su*^[b]^


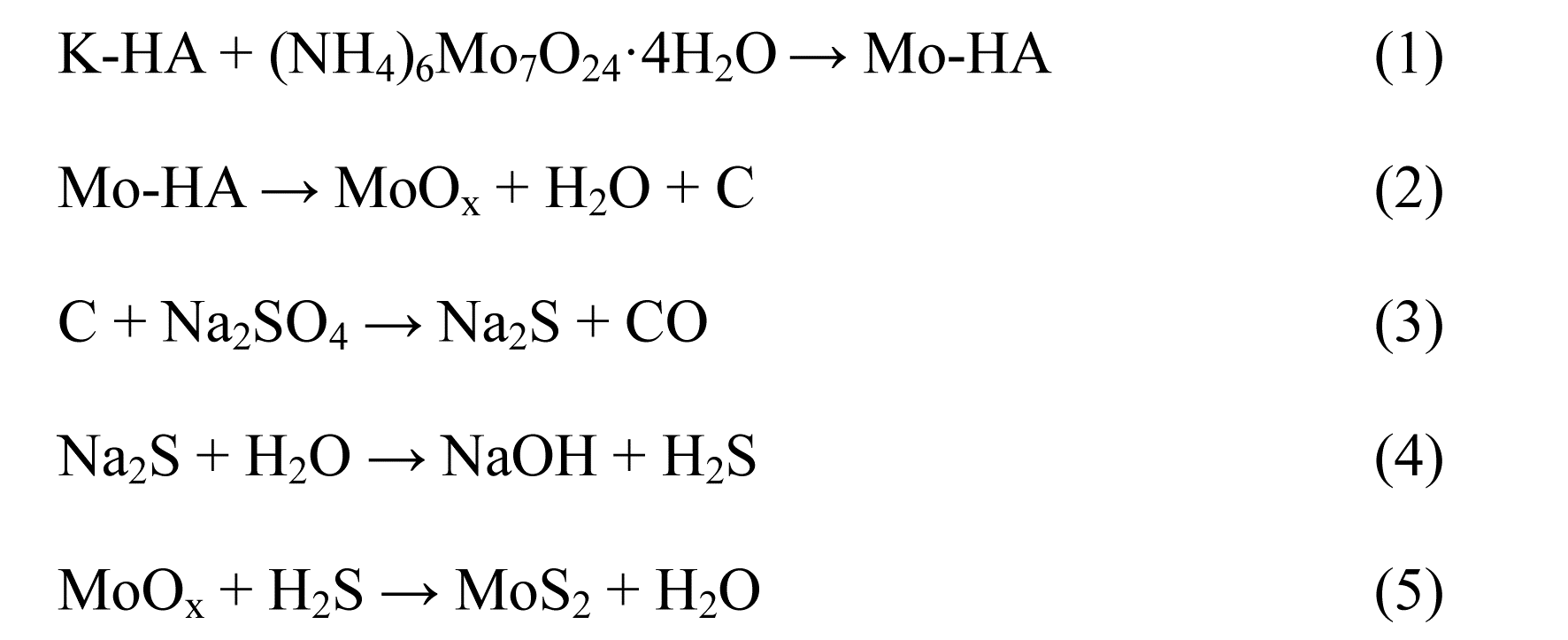


**Supplementary Equation 1-5**. The proposed reactions for the synthesis of MoS_2_.

|  | Moisture | Water-insoluble substance | Humic acid | K_2_O | Yellow humic acid | Other metals |
| --- | --- | --- | --- | --- | --- | --- |
| Content  (%) | 10.4 | 6.6 | 62 | 12.1 | 7.9 | <1 |

**Table S1.** The composition analysis of potassium humate.


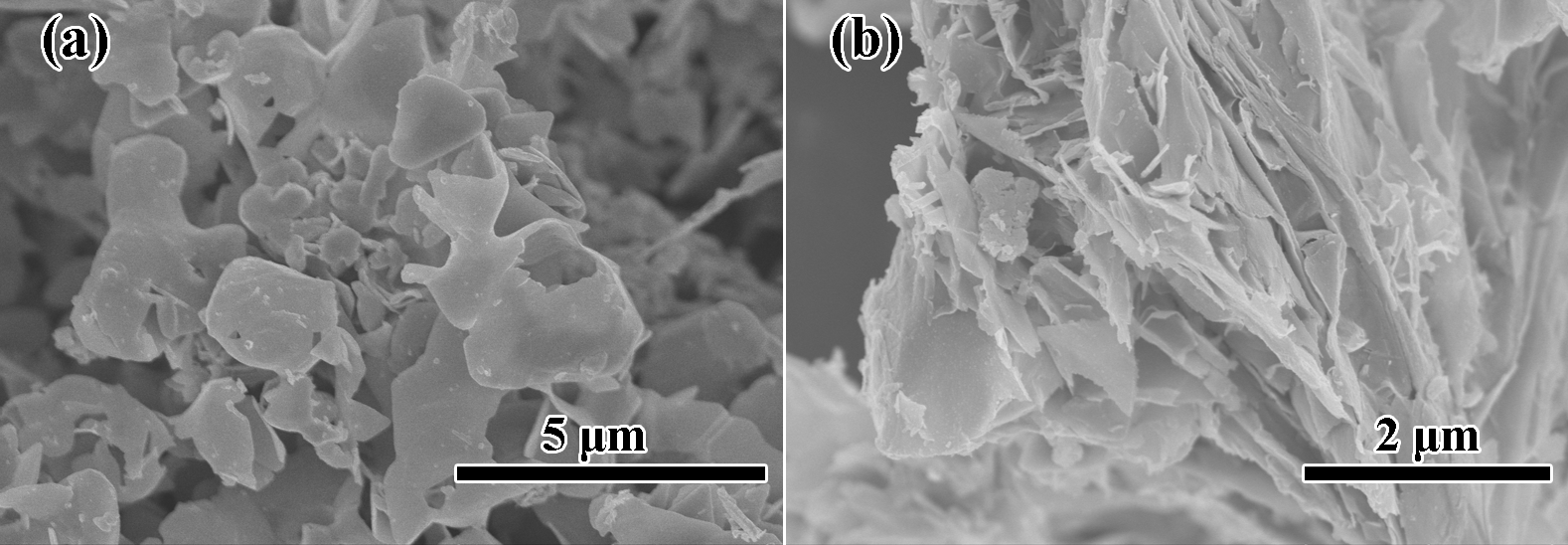


**Figure S1.** SEM images of (a) MoS_2_/C-600 and (b) MoS_2_/C-800 nanocomposite.

**
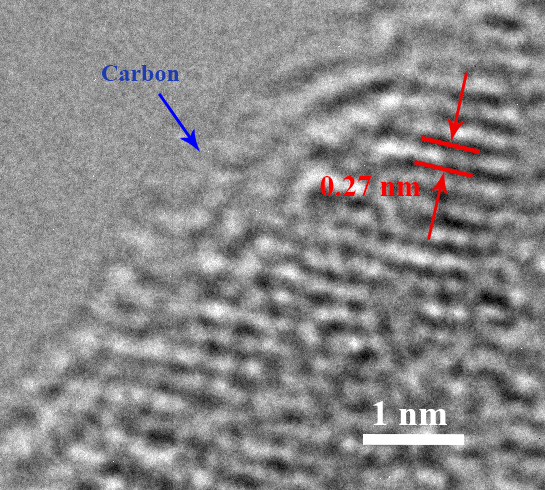
**

| Material | Current density  (mA g^-1^) | Discharge Capacities  after 50 cycles (mAh g^-1^) | Reference |
| --- | --- | --- | --- |
| MoS_2_  MoS_2_/PAIN-1  Solid MoS_2_ nanoparticles  MoS_2_/C composite  MoS_2_/C-700 | 100 | 500  580  590  400  554.9 | Present work |

**Figure S2.** High resolution TEM image of MoS_2_/C-700 nanocomposite.

**Table. S2** Comparison of electrochemical performance of MoS_2_-based electrodes.

**References**

1. Wang Z, Chen T, Chen W, Chang K, Ma L, Huang G, Chen D, Lee J (2013**)** CTAB-assisted synthesis of single-layer MoS_2_-graphene composites as anode materials of Li-ion batteries. J. Mater. Chem. A 1:2202-2210.

2. Yang L, Wang S, Mao J, Deng J, Gao Q, Tang Y, Schmidt OG (2013**)** Hierarchical MoS_2_/polyaniline nanowires with excellent electrochemical performance for lithium-ion batteries. Adv. Mater. 25:1180-1184.

3. Wang M, Li G, Xu H, Qian Y, Yang J (2013**)** Enhanced lithium storage performances of hierarchical hollow MoS_2_ nanoparticles assembled from nanosheets. ACS Appl. Mater. Interfaces 5:1003.

4. Zhou J, Qin J, Zhang X, Shi C, Liu E, Zhao N, He C (2015**)** 2D Space-Confined Synthesis of Few-Layer MoS_2_ Anchored on Carbon Nanosheet for Lithium-Ion Battery Anode. ACS Nano 9:3837.
